# The first complete mitogenome of Indian ringneck *(Psittacula krameri)* demonstrates close phylogenetic relationship with Eclectus parrot

**DOI:** 10.1080/23802359.2019.1676676

**Published:** 2019-10-15

**Authors:** Subir Sarker, Michelle Sutherland, Saranika Talukder, Shubhagata Das, Jade K. Forwood, Karla Helbig, Shane R. Raidal

**Affiliations:** a Department of Physiology, Anatomy and Microbiology, School of Life Sciences, La Trobe University, Melbourne, Australia;; b The Unusual Pet Vets, Frankston, Australia;; c School of Agriculture and Food, Faculty of Veterinary and Agricultural Sciences, The University of Melbourne, Victoria, Australia;; d School of Biomedical Sciences, Faculty of Science, Charles Sturt University, >Wagga Wagga, Australia;; e School of Animal and Veterinary Sciences, Faculty of Science, Charles Sturt University, Wagga Wagga, Australia

**Keywords:** Avian mtDNA, mitogenome phylogeny, family Psittacidae, *Psittacula krameri*

## Abstract

This study was aimed to sequence the first complete mitochondrial genome from an Indian ringneck parrot (*Psittacula krameri*). The mitogenome sequence was circular and 16,413 bp in length. In comparison to other available mitogenome sequences belonging to *Psittacidae* species, this mitogenome encoded a conserved structure consisting of 13 protein-coding genes (PCGs), two rRNA genes, 21 tRNA genes and a control region; however, this mitogenome missing a tRNA-Glu. The lengths of 12S and 16S ribosomal RNA were 975 bp and 1582 bp, respectively. The overall base composition of the mitogenome of *P. krameri* was dominated by higher AT (53.5%) than GC (46.5%) content. The complete mitogenome sequence determined in this study would be useful to track the more profound evolutionary history and the conservation of *P. krameri.*

The Indian ringneck, also known as Indian ring-necked parakeet (*Psittacula krameri*), is a pale yellow-green with a distinguishing long-tail parrot under the genus *Psittacula*, which comprises 14–15 species and 17–18 subspecies (Groombridge et al. 2004). The Indian ringneck lives in tropical and subtropical lightly wooded habitats in Africa and Asia (Latitude 2011). It is one of the most widely introduced parrots in the world, and the species do not appear to be threatened globally, and therefore it is listed as least concern by the IUCN. However, it is recorded by Ghana in Appendix III to the Convention on International Trade in Endangered Species of Wild Flora and Fauna and is currently listed as a controlled animal in Tasmania under the Nature Conservation Act 2002 (Latitude 2011). To evaluate the process of diversity and evolutionary relationships among the majority of *Psittacula* species including Indian ringneck, a molecular phylogeny was constructed using partial mitochondrial DNA sequence of cytochrome *b* gene, and demonstrated that the Indian ringneck is the most recent common ancestor of the echo parakeet on Mauritius (Groombridge et al. 2004). However, we believe that the complete mitogenome could play a significant role to provide more clear evolutionary relationships, and divergence time of speciation, as well as influencing conservation and management decisions of species. Therefore, this study was designed for sequencing a complete mitogenome of *P. krameri,* which will further strengthen our understanding of the species diversity, host phylogeny and ecological diversity of the species.

The lung tissue sourced from a deceased juvenile Indian ringneck (*P. krameri*) in a captive aviary flock in Victoria was used in this study (year of sampling: 2016; GPS location: Latitude: 37°50′59.28″S, Longitude: 145°07′8.4″E). The sample was stored in appropriate conditions by the Veterinary Diagnostic Laboratory (VDL), Charles Sturt University under the accession number CS16-4047. Animal sampling was obtained in accordance with approved guidelines set by the Australian Code of Practice for the Care and Use of Animals for Scientific Purposes (1997) and approved by the Charles Sturt University Animal Ethics Committee (Research Authority permit 09/046), and the total genomic DNA was extracted using an established protocol (Sarker, Das, et al. 2017; Sarker, Das, et al. 2018). The genomic library preparation and sequencing was performed according to the published protocol (Sarker, Roberts, et al. 2017; Sarker, Isberg, et al. 2018; Sutherland et al. 2019). Briefly, the paired-end library with an insert size of 301 bp was prepared using the Illumina Nextera XT DNA Library Prep V3 Kit (Illumina^®^ Inc., San Diego, CA) according to the manufacturer's instructions. Cluster generation and sequencing of the DNA-library was executed on Illumina^®^ MiSeq chemistry according to the manufacturer’s instructions, which was generated approximately 7.2 million reads. The raw datasets were trimmed to pass the quality control based on PHRED score or per base sequence quality score, and the assembly of the mitochondrial genome was conducted using SPAdes assembler (version 3.10.0) (Bankevich et al. 2012) in Geneious.

Annotation was performed with MITOS (Bernt et al. 2013), and protein-coding ORFs were further assessed using Geneious (version 10.2.2).

The complete mitogenome sequence of *P. krameri* had a circular genome of 16,413 bp, containing 13 protein-coding genes (PCGs), two rRNA genes, 21 tRNA genes and a control region; however, the mitogenome was missing a tRNA-Glu. The contents of A, T, C and G were 31.0%, 22.5%, 32.9% and 13.6%, respectively. AT and GC contents of this complete mitogenome was 53.5% and 46.5%, respectively. The proportion of coding sequences with a total length of 11,068 bp (67.43%), which encodes 3676 amino acids, and all protein-coding genes started with Met. The lengths of 12S and 16S ribosomal RNA were 975 bp and 1582 bp, respectively. The gene arrangement was similar to the complete mitochondrial genome of other *Psittacidae* species.

The complete mitogenome sequence of a *P. krameri* determined in this study, and other mitogenome sequences belong to the family *Psittacidae* were retrieved from NCBI database, and were aligned using the MAFFT L-INS-i algorithm in Geneious (Katoh et al. 2002). A maximum-likelihood (ML) phylogenetic tree with 1000 non-parametric bootstrap resamplings was generated using Geneious (version 10.2.2). As highlighted in Figure 1, the mitogenome sequence of *P. krameri* produced a monophyletic clade with Eclectus parrot (*Eclectus roratus*; GenBank accession no. KM611469) (Eberhard and Wright 2016), and demonstrated a > 90% pairwise nucleotide identity between them. We concluded that the complete mitogenome of *P. krameri* will be a useful database among the genus *Psittacula* to study the further host-phylogenetic relationship of *Psittacula* species, and suggests that this may be an implication for the conservation and management of the species.

**Figure 1.**
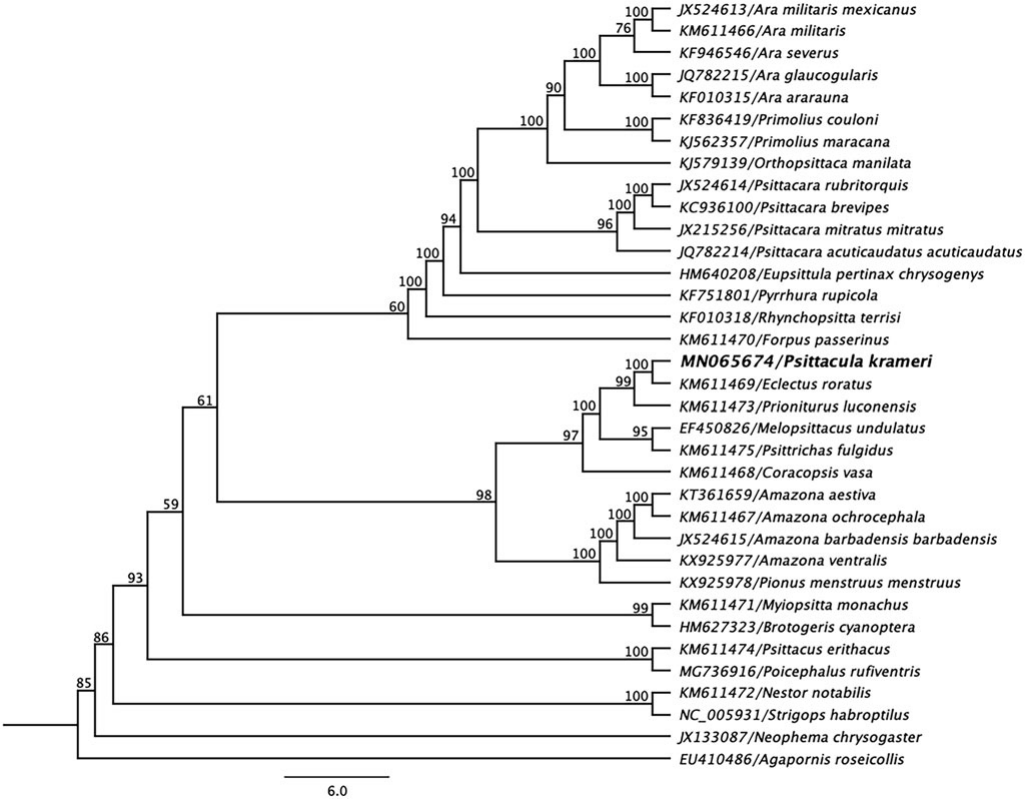
Maximum-likelihood phylogenetic tree to infer host-phylogeny relationship among *Psittacidae* family. ML-tree was constructed using complete mitogenome sequences of the species belongs to the *Psittacidae* family. The new complete mitogenome of *P. krameri* was highlighted by bold font.
